# MicroRNA expression analysis of feline and canine parvovirus infection in vivo (felis)

**DOI:** 10.1371/journal.pone.0185698

**Published:** 2017-10-19

**Authors:** Pei Zhou, Xin Zhang, Weijie Zeng, Qingxu Zheng, Xiangqi Hao, Xi Lin, Yun Zheng, Lifang Wang, Guihong Zhang, Shoujun Li

**Affiliations:** 1 College of Veterinary Medicine, South China Agricultural University, Guangzhou, People’s Republic of China; 2 Key Laboratory of Comprehensive Prevention and Control for Severe Clinical Animal Diseases of Guangdong Province, Guangzhou, People’s Republic of China; 3 Guangdong Engineering and Technological Research Center for Pets, Guangdong, Guangzhou, People’s Republic of China; University of British Columbia, CANADA

## Abstract

Feline panleukopenia is a common contagious disease with high morbidity and mortality. At present, feline parvovirus (FPV) and canine parvovirus (CPV) variants are the pathogens of feline panleukopenia. Many studies have shown that miRNAs are involved in virus-host interactions. Nevertheless, miRNA expression profiling of FPV (original virus) or CPV-2b (new virus) in cats has not been reported. To investigate these profiles, three 10-week-old cats were orally inoculated with 10^6^ TCID_50_ of the viruses (FPV and CPV-2b), and the jejunums of one cat in each group were sectioned for miRNA sequencing at 5 days post-inoculation (dpi). This study is the first attempt to use miRNA analysis to understand the molecular basis of FPV and CPV infection in cats. The miRNA expression profiles of the jejunums of cats infected with FPV and CPV were obtained, and a subset of miRNAs was validated by real-time qPCR. The results show that a variety of metabolism-related pathways, cytokine- and pathogen-host interaction-related pathways, and pathology- and cellar structure-related pathways, as well as others, were affected. Specifically, the JAK-STAT signaling pathway, which is critical for cytokines and growth factors, was enriched. This description of the miRNAs involved in regulating FPV and CPV infection in vivo provides further insight into the mechanisms of viral infection and adaptation and might provide an alternative antiviral strategy for disease control and prevention.

## Introduction

Feline panleukopenia, a common contagious disease with high morbidity and mortality, is caused by the single-stranded DNA virus feline parvovirus (FPV). FPV is a member of the genus *Protoparvovirus* in the family *Parvoviridae*. The DNA genome of FPV is 5.2 kb long and contains two open reading frames (ORFs). The first ORF encodes two non-structural proteins, NS1 and NS2, and the second ORF encodes two structural proteins, VP1 and VP2 [1]. Canine parvovirus (CPV) derived from FPV with several amino acid mutations [2–6], but other evidence suggests that CPV and FPV might have evolved separately from common viral ancestors in wildlife [7]. The virus was first named CPV-2 to distinguish it from the unrelated canine minute virus (CMV) [8]. In 1978, the original CPV-2 was first identified in canines in the United States [9], and it then quickly became widespread, with outbreaks in many countries [10]. With virus evolution, the novel antigenic variants CPV-2a/2b/2c have been reported around the world [11, 12].

MicroRNAs (miRNAs) are endogenous small noncoding RNAs of approximately 18–23 nucleotides (nt) in length. miRNAs play a critical role in many biological processes in both plants and animals via two mechanisms [13–15]. In plants, miRNAs are precisely or nearly precisely complementary to their target mRNAs, and the mRNAs are cleaved [16]. In animals, miRNAs are generally imprecisely complementary to their mRNA targets, leading to the translational repression or degradation of the target mRNAs [17].

Initially, FPV could replicate within the thymus but not within the intestinal tract of dogs and was therefore not shed [18, 19], and the original CPV-2 did not infect cats [20]. However, the CPV-2a/2b/2c variants that are naturally isolated from cats could experimentally infect cats [20–22]. Moreover, CPV-2 variants caused up to approximately 80% of cases of feline panleukopenia in unvaccinated cat populations based on one report in Vietnam and Taiwan [21]. In addition, the phenomenon of asymptomatic infection by CPV-2 variants in cats has been characterized by Clegg et al. [23]. Nevertheless, miRNA expression profiling in FPV or CPV infection of cats has not been reported. In this study, HiSeq2500 was used to sequence differentially expressed miRNAs in infected and non-infected cat jejunums, and the corresponding expression levels associated with FPV infection and CPV infection were analyzed.

## Material and methods

### Virus isolation

CPV (CPV-2-G1, accession number: KY418607) was isolated from a dog (golden retriever breed, 4 months age), and FPV (FPV-G1, accession number: KY451727) was isolated from a cat (domestic short-hair breed, 5 months age) in 2016. In this etiological investigation, anal swabs of animals were collected in veterinary clinics in Guangzhou, Guangdong, China. All animals had clinical signs of parvovirus infection. The samples were preserved at -80°C. This study protocol was reviewed and approved by the animal welfare ethics committee. The detection PCR primers CPV-F (5’-TGATTGTAAACCATGTAGACTAAC-3’) and CPV-R (5’-TAATGCAGTTAAAGGACCATAAG-3’) were used for virus detection. The PCR amplification process was 35 cycles of 94°C for 1 min, 55°C for 30 s, and 72°C for 1 min, with a final extension at 72°C. A PCR product size of approximately 567 bp was considered positive for CPV. The virus was isolated and propagated in F81 cells and titrated using TCID_50_/ml.

### Animal experiments

Nine 10-week-old, domestic short-hair breed, and specific pathogen-free (SPF) cats were purchased and housed in certified BSL-2 facilities at South China Agricultural University. Prior to commencement, anal swabs and serum samples were collected and tested. The virus detection and serological assays were performed to ensure that animals were negative for FPV, CPV and other viruses. Viruses were detected using an antigen test strip kit (*South Korea*, *BioNote*, *Inc*. *Anigen rapid CPV Ag test kit*, *CAT*. *No*.: *RG11-01*), PCR detection (forward primer: F-5’ TGATTGTAAACCATGTAGACTAAC3’ reverse primer: R-5’TAATGCAGTTAAAGGACCATAAG3’) and virus isolation from F81 cells. The antibody detection procedure used the antibody test strip kit (*South Korea*, *BioNote*, *Inc*. *Anigen rapid CPV Ab test kit*, *CAT*. *No*.: *RB21-51*) and an indirect immunofluorescent assay (IFA). Three cats per group were orally inoculated with 10^6^ TCID_50_ FPV or CPV in 1.0 ml of phosphate-buffered saline (PBS). As a control, an additional three cats were orally inoculated with 1.0 ml of PBS. All animals were monitored daily for clinical signs, and anal swabs were collected for virus shedding and analysis by TCID_50_/ml. At 5 dpi, one cat from each inoculation group and the control group were euthanized by an intravenous injection of pentobarbital, and necropsies were performed. The brain, heart, liver, spleen, lung, kidney, stomach, duodenum, jejunum, ileum, cecum, and colon were collected to evaluate viral replication. The jejunums were sectioned for histopathological and immunochemical examination and miRNA sequencing. For histopathological and immunochemical examination, tissue sections were fixed in 10% phosphate-buffered formalin. Twenty-four hours later, the tissues were dehydrated, embedded in paraffin, and cut into 5-μm-thick sections before being stained with hematoxylin-and-eosin (H&E, Sigma). Standard immunohistochemistry (IHC) assays were performed by incubating the sections with a mouse monoclonal antibody raised against the influenza A virus nucleoprotein (Sigma) and then with horseradish peroxidase (HRP)-conjugated goat anti-mouse IgG (H+L) antibody (Abbkine), and the sections were visualized with diaminobenzidine (DAB).

### Ethics statement

All procedures in the virus isolation and animal experiments met the requirements and were approved by the Experimental Animal Welfare Ethics Committee of the South China Agricultural University. According to the requirements, euthanasia was carried out when the cats showed severe dehydration, complete anorexia and when they were unable to stand up. Euthanasia was performed using an intravenous injection of pentorbarbital. All experimental animals were monitored by university-licensed veterinarians.

### RNA preparation and sequencing

The sectioned jejunum samples were flash frozen in liquid nitrogen and stored at -80°C until RNA processing. Total RNA was extracted from the inoculated and control tissues using the Trizol reagent (Invitrogen, USA) according to the manufacturer’s protocol. The cDNA library was established according to the protocol of the NEB Next Ultra Small RNA Sample Library Prep Kit by Illumina. Briefly, 3’ and 5’ adaptor ligation was followed by first-strand cDNA synthesis. PCR enrichment was followed by clean up and size selection. After library establishment, the concentration of the library was measured using a Qubit 2.0 (Life Technologies). The concentration of the library was diluted to 1 ng/μl. The insert size was measured with an Agilent 2100 bioanalyzer [24]. To ensure quality, the q-PCR method was used to quantify the concentration of the library. Finally, Illumina HiSeq2500 was used for high-throughput sequencing, and the sequencing read length was set to single-end (SE) 50 nt.

### Bioinformatics analysis

#### Sequence clean-up

To ensure the accuracy of the information analysis, we controlled the quality of the original data and the high-quality sequences (clean reads). The standard of the original sequence quality control was as follows: remove low-quality sequences; remove sequences with unknown base content greater than or equal to 10% reads; remove reads with no 3’ adaptor sequence or no insert sequence; remove the 3 'adapter sequences’; and remove sequences with fewer than 18 or more than 30 nucleotides. The clean reads were compared with the Silva, GtRNAdb, Rfam and Repbase databases using Bowtie [25] to filter the ribosomal RNA (rRNA), transfer RNA (tRNA), intranuclear small RNA (snRNA), small nucleolar RNA (snoRNA) and repeat sRNA and to obtain the unannotated reads, which were from miRNAs. Unannotated reads were aligned with the *Felis catus* genome (ftp://ftp.ensembl.org/pub/release-84/fasta/felis_catus/dna/) using miRDeep2 [26] to map them to the genome.

#### miRNA analysis

The miRNAs were identified using miRDeep2, which is mainly used for animal miRNA prediction [26]. The miRNAs were aligned with the miRNAs of all reference species in miRBase 21 to identify homologs of the miRNAs. miRNA expression was statistically analyzed and was normalized using the TPM algorithm [27].

#### miRNA differential expression analysis

DESeq [28] was used in this study for differential expression analysis between the sample groups. The fold change (FC) represents the ratio of expression between two groups. The p-value of the original hypothesis is expressed as the probability of non-discrepancy. Because miRNA differential expression analysis requires independent statistical hypothesis tests for the expression of a large number of miRNAs, there is an increased likelihood of false positives; therefore, in the analysis process, the Benjamini-Hochberg correction method was used to test the significance of the original hypothesis p-value, and the false discovery rate (FDR) was used as the key indicator for the screening of differentially expressed miRNAs. |log2 (FC) |> = 1 and FDR < = 0.05 were the screening criteria used in this analysis.

#### Target gene prediction and KEGG annotation

According to the obtained miRNA sequences and how they correspond to the felis genome, target gene prediction was conducted using the special animal target gene prediction programs miRnada [29] and RNAhybrid [30]. Then, the genes from the intersection of those from miRnada and RNAhybrid were used for the annotation. To obtain information on the Kyoto Encyclopedia of Genes and Genomes (KEGG) annotation of the target genes, the predicted target gene sequences were compared with the KEGG database using BLAST software. The network of miRNAs, target genes, and KEGG annotations was established using Gephi 0.9.1, and the annotation map was downloaded from the website http://www.kegg.jp/kegg-bin/show_pathway?fca04630.

#### Real-time qPCR

Approximately 20-200-nt small RNA samples were isolated using the miRcute miRNA isolation kit (Tiangen Bio.Tech. Co., Ltd. Product no: DP501), the cDNA synthesis was performed using a miRcute plus miRNA first-strand cDNA synthesis kit (Tiangen Bio.Tech. Co., Ltd. Product no: KP211), and the qPCR reactions used a miRcute plus miRNA qPCR detection kit (Tiangen Bio.Tech. Co., Ltd. Product no: FP411). Subsequently, real-time qPCR was performed using a standard protocol on a Roche LightCycler® 480 Instrument II. Briefly, after initial denaturation at 95°C for 30 s, amplification was conducted for 40 cycles with denaturation at 95°C for 10 s, primer annealing at 60°C for 10 s, and DNA extension at 72°C for 10 s. All samples were run in triplicate, and the amount of each miRNA relative to that of U6 RNA was calculated using the 2^-△△Ct^ method [31]. Primers are listed in S1 Table.

## Results

### Experimental infection in cats

**Fig 1:**
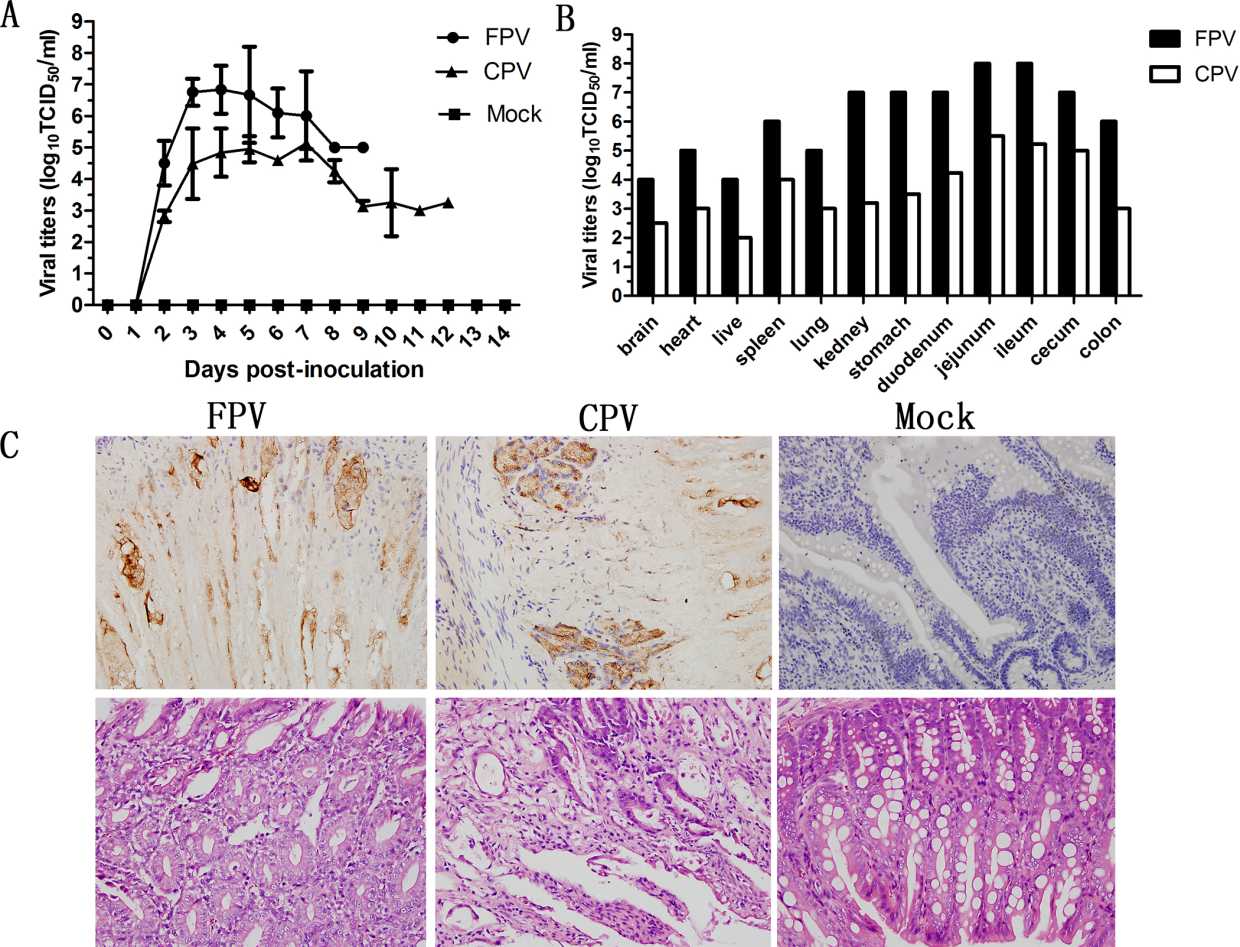
The pathology of FPV and CPV-2b experimental infection in cats. A: Virus shedding in inoculated cats. B: Viral replication in tissues of inoculated cats that were euthanized on 5 dpi. C: Histological examination of infected cat jejunums.

Amino acid 426 of VP2 of CPV-2-G1 is aspartic acid (D), which indicates that the virus belongs to CPV-2b. Both the FPV- and CPV-inoculated cats showed severe diarrhea and hematochezia. Moreover, in addition to the one euthanized cat in each group, the other two FPV-inoculated cats died at 7 dpi and 9 dpi, and the other two CPV-inoculated cats died at 10 dpi and 11 dpi. Anal swabs were collected starting at 1 dpi and continued until death. All inoculated cats persistently shed the virus from 2 dpi until death, and viral titers were persistently near 7.0 logTCID_50_/ml in the FPV group and near 5.0 logTCID_50_/ml in the CPV group (Fig 1A). Viral replication was detected in all tissues (brain, heart, liver, spleen, lung, kidney, stomach, duodenum, jejunum, ileum, cecum, and colon) after the cats were euthanized, and the viral titers were 4.0 to 8.0 logTCID_50_/ml in the FPV group and 2.0 to 5.5 logTCID_50_/ml in the CPV group (Fig 1B). The viral replication in the jejunum and ileum was the highest (Fig 1B), indicating severe macroscopic pathological lesions. Histologically, viral antigen expression was detected in the jejunal tissues of FPV-and CPV-inoculated cats (Fig 1C). The H&E staining of the jejunal tissues showed evidence of cell collapse and massive lymphocyte recruitment (Fig 1E). The results confirm that both FPV and CPV-2b can cause lethal systemic infection in cats after experimental inoculation. Viruses replicate at a high titer and cause severe lesions within the jejunum.

### miRNA identification

The transcription initiation sites of miRNAs are mainly located in the intergenic regions, introns, and reverse complementary sequences of the coding sequence. The precursor of miRNAs has a marked hairpin structure. The mature miRNA is produced by Dicer/DCL enzyme cleavage [14, 17]. Herein, miRDeep2 was used to identify miRNAs for alignment to the reference genome. There are 1038, 976 and 1036 miRNAs predicted in mock jejunums, FPV jejunums and CPV jejunums, respectively. The total number of miRNAs in all samples was 1075. miRNAs are highly conserved among species, and the miRNAs were analyzed for their miRNA family evolution based on the similarity of sequences. According to the analysis, there are 456 miRNAs that could align to the other families and 619 unique miRNAs for felis catus.

### Analysis of differentially expressed miRNAs

**Fig 2:**
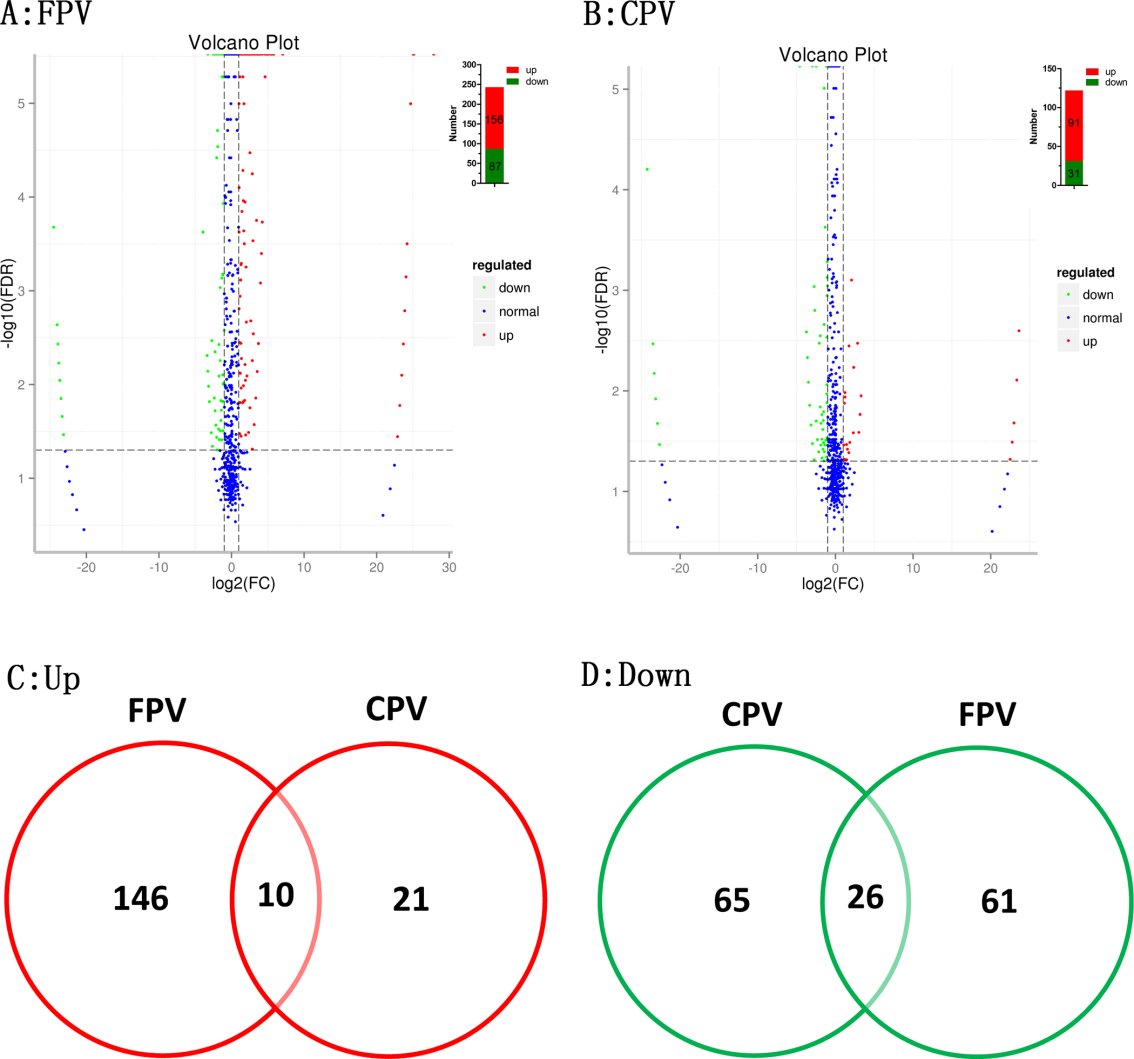
The difference of miRNA expression levels. A: Statistical significance of the differences in FPV infection in jejunums is showed in the volcano plot. The top right corner shows the number of differentially expressed miRNAs. B: Statistical significance of the differences in CPV infection in jejunums is showed in the volcano plot. The top right corner shows the number of differentially expressed miRNAs. Distributions of up-regulated (C) and down-regulated (D) miRNAs in FPV and CPV infection. The data depicted here is derived from one cat in each of the three groups.

The differences in the miRNA expression levels between the groups were further analyzed by DESeq. The statistical significance of the differences could be seen in the volcano plots (Fig 2A and 2B). Of the differentially expressed miRNAs, 156 were up-regulated and 87 were down-regulated in the FPV infection group (Fig 2A), and 91 were up-regulated and 31 were down-regulated in the CPV infection group (Fig 2B). There were 10 up-regulated and 26 down-regulated miRNAs in both the FPV and CPV infection groups (Fig 2C and 2D). Subsequently, the intersection of the differentially expressed miRNAs was validated by real-time qPCR. With the exception of 2 down-regulated miRNAs, the relative expression of the other 34 miRNAs was consistent with the high-throughput sequencing results (S1 Fig). The log2 FC values and sequences of these 34 miRNAs are shown in Table 1. Based on the sequences, the 34 miRNAs were temporarily termed fca-mir-1 to fca-mir-32. fca-miR-16a and fca-miR-16b are highly homologous, and fca-miR-27a-1 and fca-miR-27a-2, which are transcribed from different chromosomes, have the same mature sequence (Table 1).

**Table 1:** Summary of differentially expressed levels miRNA in FPV infection and CPV infection group. According to the descending order of the log2FC value of FPV vs Control.

ID	miRNA	Sequence	FPV vs Control (log2FC)	CPV vs Control (log2FC)	Target genes
B3_358081	fca-miR-1	cuuggucuauuuuugcucuuu	27.86060797	23.64152084	0
F2_880617	fca-miR-2	acuggaagugguuuaguagagc	23.19385138	22.76708847	3
X_900844	fca-miR-3	uucauucggcuguccagaugua	3.709741783	3.171866718	5
JH408484.1_921063	fca-miR-4	aauccugaacaacaaagc	3.539816782	3.171866718	0
B1_222146	fca-miR-5	gggggcgccuggguggcugagucg	2.955218644	1.724816203	1724
F1_849077	fca-miR-6	ugcugagucagaugcgggcu	2.347718283	2.85051552	23
A1_62152	fca-miR-7	uauauuuguagauuuauuuaug	2.125052546	1.550670262	0
F1_842340	fca-miR-8	cuggaggacuaagaaggcugaguc	1.655658362	2.07270407	136
B4_401040	fca-miR-9	cuucagcucaggucaugaccucc	1.328610785	1.24876938	76
F1_839896	fca-miR-10	uuggccuacagaaaugacagaca	1.317736666	1.743354382	4
A3_156191	fca-miR-11	ucgaggagcucacagucu	-1.182334571	-1.439262306	1
JH408902.1_924271	fca-miR-12	agggcaucuggagaacaacc	-1.211948279	-1.062305378	2
A1_66975	fca-miR-13	cggugguuucccuucccg	-1.448558059	-1.029918791	11
C1_509864	fca-miR-14	cugggagaggugggggagg	-1.547324567	-2.652159378	431
D2_612931	fca-miR-15	uaauuagccugcagugugacu	-1.682263334	-1.372060645	0
A3_149904	fca-miR-16a	ugagugugugugugugaaugu	-2.044872455	-1.412822519	4
F2_872673	fca-miR-17	aaaaaaggacaggaacgaaaca	-2.267225835	-1.635175899	0
D4_705373	fca-miR-18	gaugaggucuguguaugcu	-2.267225835	-1.957023146	1
X_910787	fca-miR-19	uuggggagaaggugguaggccgugu	-2.267225835	-1.149783722	788
A2_78409	fca-miR-20	gugagggccaggccccuggag	-2.366782331	-24.23870561	586
A2_93750	fca-miR-21	cggcccuggcggagcgcg	-2.518770848	-2.986291237	427
D3_672076	fca-miR-22	uugggagugcagcucuggcu	-2.629743857	-2.319743296	35
X_905047	fca-miR-23	uuggcuaaggcaauuuuguau	-2.629743857	-23.50166714	0
B4_408522	fca-miR-24	uagcuugacuugugcuucuc	-2.78178339	-3.471176528	0
B4_430496	fca-miR-25	ugugcaugcgugcgcgugc	-3.044872455	-1.412822519	4
D1_588871	fca-miR-26	ugugugcgggucuguccccc	-3.044872455	-1.412822519	46
A2_87326	fca-miR-27a-1	auugaugauggcuguguaguucc	-3.044872455	-1.734871895	7
C1_488789	fca-miR-27a-2	auugaugauggcuguguaguucc	-3.044872455	-1.734871895	7
E3_824821	fca-miR-16b	ugagugugugugugugggg	-3.919316728	-4.608709866	16
A2_129547	fca-mir-28	cuggggggacgcgggcgacgcu	-23.13914912	-2.956618973	1222
A3_170418	fca-mir-29	cgccccugccccgcucccc	-23.13914912	-23.13914912	409
C2_529539	fca-mir-30	uccucugaugaauacugauu	-23.13914912	-23.13914912	0
E3_817537	fca-mir-31	guggugguggacugugaguc	-23.79124001	-3.608709866	50
A2_76321	fca-mir-32	ugggaggugagagcgagugguu	-24.50172787	-2.7346394	224


ID: the chromosomes and the position on the chromosomes.

Red shade presents down-regulation and green shade presents up-regulation.

### KEGG annotation of target genes

**Fig 3:**
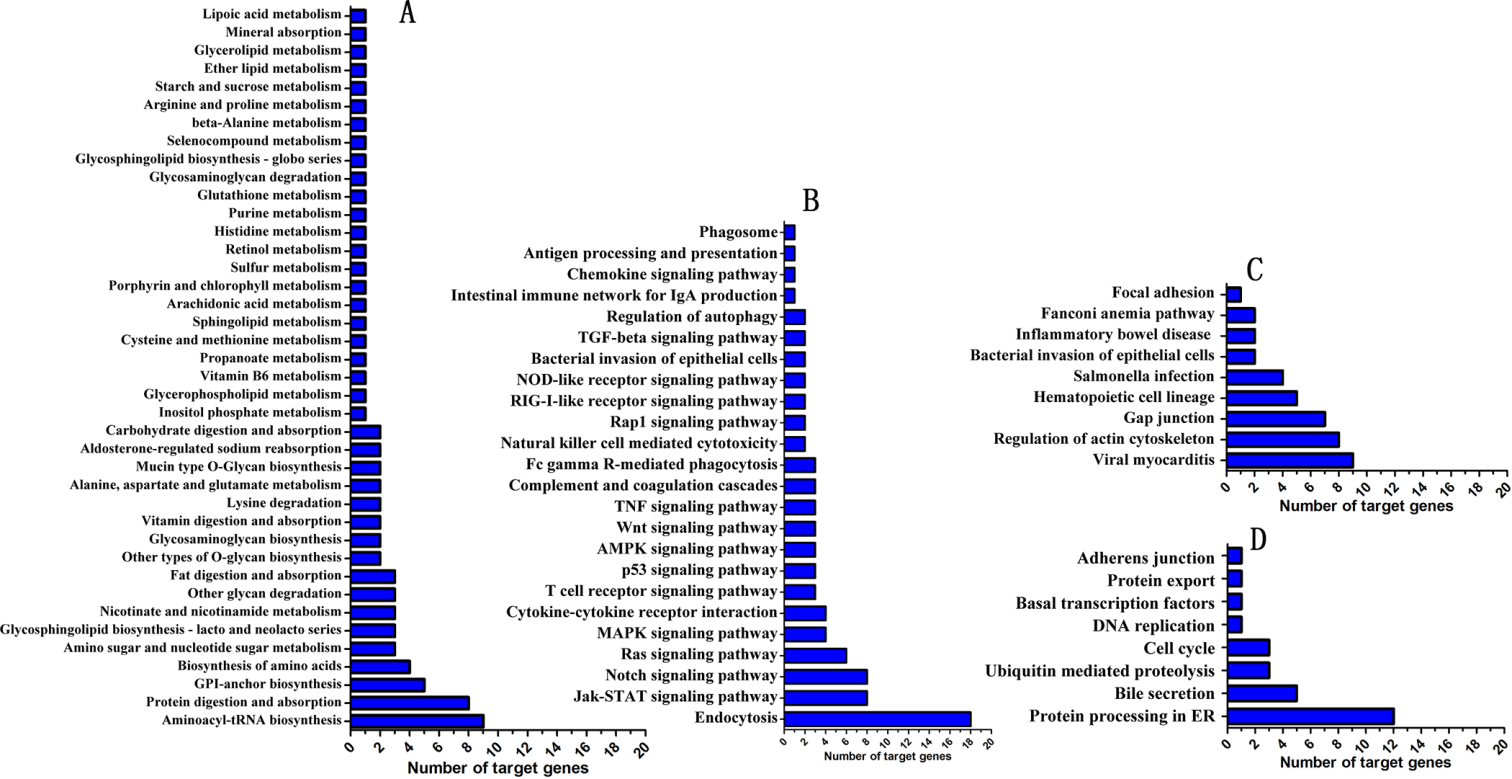
KEGG pathways of target genes of differentially expressed miRNAs. A: Metabolism-related pathways. B: Cytokine- and pathogen-host interaction-related pathways. C: Pathology and cellar structure-related pathways. D: Other pathways.

**Fig 4:**
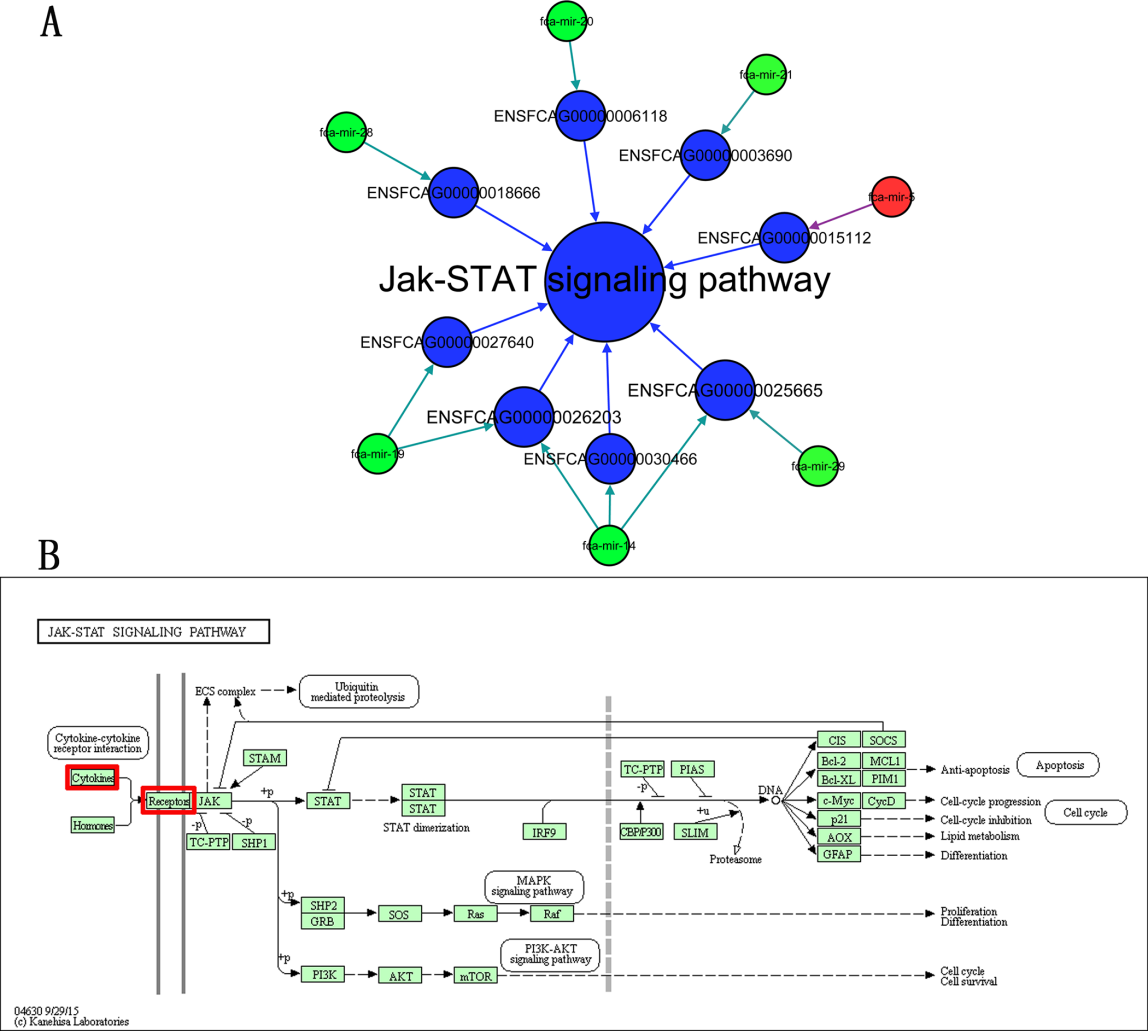
The JAK-STAT signaling pathway. A: The interaction network of the miRNAs, target genes, and JAK-STAT signaling pathway. B: Diagram of the JAK-STAT signaling pathway from the KEGG database. The red blocks mark the regulated genes.

Of the 34 miRNAs, 26 were predicted to have a total of 6242 overlapping target genes (Table 1). The sequences of the target genes were annotated with the KEGG database. After classification, there were 40 metabolism-related pathways that were annotated (Fig 3A). There were 24 pathways that were annotated as related to cytokines and pathogen-host interaction (Fig 3B), 9 pathways were annotated as pathology-related and cell structure-related (Fig 3C), and 8 pathways were annotated as others (Fig 3D). Among the cytokine- and pathogen interaction-related pathways, there were 14 significant immune system-related pathways (Fig 3B): the JAK-STAT signaling pathway, cytokine-cytokine receptor interaction, T cell receptor signaling pathway, TNF signaling pathway, complement and coagulation cascades, Fc gamma R-mediated phagocytosis, natural killer cell-mediated cytotoxicity, RIG-I-like receptor signaling pathway, NOD-like receptor signaling pathway, TGF-beta signaling pathway, regulation of autophagy, intestinal immune network for IgA production, phagosome, and antigen processing and presentation. The JAK-STAT signaling pathway, which is critical for cytokines and growth factors, was further analyzed. A total of 6 down-regulated miRNAs (fca-mir-14, fca-mir-19, fca-mir-20, fca-mir-21, fca-mir-28, fca-mir-29) and 1 up-regulated miRNA (fca-mir-5) affect the JAK-STAT pathway through the targeting of 8 genes (Fig 4A) that encode cytokines and cytokine receptors (Fig 4B).

## Discussion

miRNAs play a critical role in many biological processes by mediating the translational repression or degradation of target mRNAs in animals [17]. It is well known that virus replication depends on the host cellular machinery. Additionally, many studies have shown that miRNAs are involved in virus-host interactions. Host miRNAs have direct antiviral effects on the primate foamy virus type 1 retrovirus [32], human immunodeficiency virus [33], influenza A virus [34], and hepatitis C virus [35]. The host miRNAs affect antiviral innate immune responses to virus infection [36–39], and virus-encoded miRNAs regulate interactions between viruses and hosts [40–42]. MicroRNAs being involved in virus-host interactions might provide us with an alternative strategy for disease prevention and control, like developing the direct antiviral effects, and stimulating the antiviral innate immune responses to virus infection. To date, FPV and CPV have been the defining pathogens for feline panleukopenia. Our results confirm the findings of others [20, 43] that CPV-2b can cause lethal systemic infections in cats resulting in high viral loads and severe segmental enteritis, predominantly affecting the jejunum. Deep sequencing was performed to explore the miRNAs that are involved in regulating this virus-host interaction model.

In the cat model, 1075 miRNAs were identified, and 34 interesting miRNAs were submitted to miRBase 21, which shrinks the gap in the felis miRNA spectrum and can benefit future miRNA research on the felis organism.

In the KEGG annotations, metabolic-related pathways were the most common. Metabolism is the basic activity of cells, and it is affected by the FPV and CPV-2b infection, which might significantly affect cellular survival via miRNA regulation.

The impact of the pathology- and cell structure-related pathways was demonstrated by the cells that were severely damaged by the viruses. Among them, the viral myocarditis pathway might be associated with some cases of CPV infection and myocardial diseases in neonatal puppies [44]. In eukaryotic cells, the actin cytoskeleton plays a crucial role in a variety of essential biological processes. For example, the actin cytoskeleton provides a structural framework for cell shape and polarity, and its dynamic properties provide the driving force for cells to divide and move [45]. Therefore, these biological processes might be severely affected by regulation of the actin cytoskeleton pathway and might result in an attenuated and flattened epithelium with shortened intestinal villi leading to the loss of osmotic regulation. Bacterial infection increases the severity of the disease, which might be consistent with the salmonella infection pathway and bacterial invasion of epithelial cell pathway, which were affected by miRNA regulation.

There are 14 significant immune system-related pathways in the cytokine and pathogen-host interaction related pathways group. Among them, the JAK-STAT signaling pathway was the most enriched pathway, and this pathway is critical for cytokines and growth factors, which are important in immune development, hematopoiesis, sexually dimorphic growth, adipogenesis, mammary gland development and lactation, and other processes [46]. A variety of ligands and their receptors stimulate the JAK-STAT pathway. In this study, the cytokines and cytokine receptors (Fig 4B) of the JAK-STAT pathway might be significantly affected by miRNA regulation of FPV and CPV infection. Specifically, the interferon (IFN)-mediated innate immune response exerts its function through the transducer and activator of the JAK-STAT pathway, which leads to a remarkable antiviral effect [47]. Therefore, the IFN-mediated innate immune response might be significantly affected by miRNA regulation in FPV and CPV infection.

This study is the first attempt to use miRNA analysis to understand the molecular basis of an original virus (FPV) and new virus (CPV) in a cat model. However, there are two potential limitations that should be considered. First, although the downstream functional analysis used qPCR to verify the miRNAs, only one sample was used for miRNA identification in FPV- and CPV-infected cats, which could introduce sampling bias into the analysis. Second, miRNA regulation of the JAK-STAT pathway needs to be verified, and the mechanism of these effects needs to be explored further.

## Supporting information

S1 Table Primer sequences used for real-time qPCR. (DOCX)

S1 Fig Validation of regulated miRNAs by real-time qPCR. The relative expression was calculated using the 2^-△△Ct^ method, and values greater than 1000 or less than -1000 are presented as 1000 or -1000.
(TIF)
