# Supplementary material for: MicroRNA expression analysis of feline and canine parvovirus infection in vivo (felis)
Source: PLoS One. 2017 Oct 19;12(10):e0185698. doi: 10.1371/journal.pone.0185698 (PMC5648106; doi:10.1371/journal.pone.0185698)
Supplement: S1 Table — (DOCX) [file pone.0185698.s001.docx]

**S1 Table** Primer sequences used for real-time qPCR.

| ID name | Sequence (5’-3’) |
| --- | --- |
| B3_358081 | F: cttggtctatttttgctcttt |
| F2_880617 | F: actggaagtggtttagtagagc |
| X_900844 | F: ttcattcggctgtccagatgta |
| JH408484.1_921063 | F: aatcctgaacaacaaagc |
| B1_222146 | F: gggggcgcctgggtggctgagtcg |
| F1_849077 | F: tgctgagtcagatgcgggct |
| A1_62152 | F: tatatttgtagatttatttatg |
| F1_842340 | F: ctggaggactaagaaggctgagtc |
| B4_401040 | F: cttcagctcaggtcatgacctcc |
| F1_839896 | F: ttggcctacagaaatgacagaca |
| A3_156191 | F: tcgaggagctcacagtct |
| JH408902.1_924271 | F: agggcatctggagaacaacc |
| A1_66975 | F: cggtggtttcccttcccg |
| C1_509864 | F: ctgggagaggtgggggagg |
| D2_612931 | F: taattagcctgcagtgtgact |
| A3_149904 | F: tgagtgtgtgtgtgtgaatgt |
| F2_872673 | F: aaaaaaggacaggaacgaaaca |
| D4_705373 | F: gatgaggtctgtgtatgct |
| X_910787 | F: ttggggagaaggtggtaggccgtgt |
| A2_78409 | F: gtgagggccaggcccctggag |
| A2_93750 | F: cggccctggcggagcgcg |
| D3_672076 | F: ttgggagtgcagctctggct |
| X_905047 | F: ttggctaaggcaattttgtat |
| B4_408522 | F: tagcttgacttgtgcttctc |
| B4_430496 | F: tgtgcatgcgtgcgcgtgc |
| D1_588871 | F: tgtgtgcgggtctgtccccc |
| A2_87326 | F: attgatgatggctgtgtagttcc |
| C1_488789 | F: attgatgatggctgtgtagttcc |
| A2_76899 | F: ggcgacgctgttgttttt |
| JH412698.1_928245 | F: gctgtgtgcatgctgagc |
| E3_824821 | F: tgagtgtgtgtgtgtgggg |
| A2_129547 | F: ctggggggacgcgggcgacgct |
| A3_170418 | F: cgcccctgccccgctcccc |
| C2_529539 | F: tcctctgatgaatactgatt |
| E3_817537 | F: gtggtggtggactgtgagtc |
| A2_76321 | F: tgggaggtgagagcgagtggtt |
| U6 | F: ctcgcttcggcagcaca |
