# Supplementary figures and images for: MicroRNA expression analysis of feline and canine parvovirus infection in vivo (felis)
Source: PLoS One. 2017 Oct 19;12(10):e0185698. doi: 10.1371/journal.pone.0185698 (PMC5648106; doi:10.1371/journal.pone.0185698)

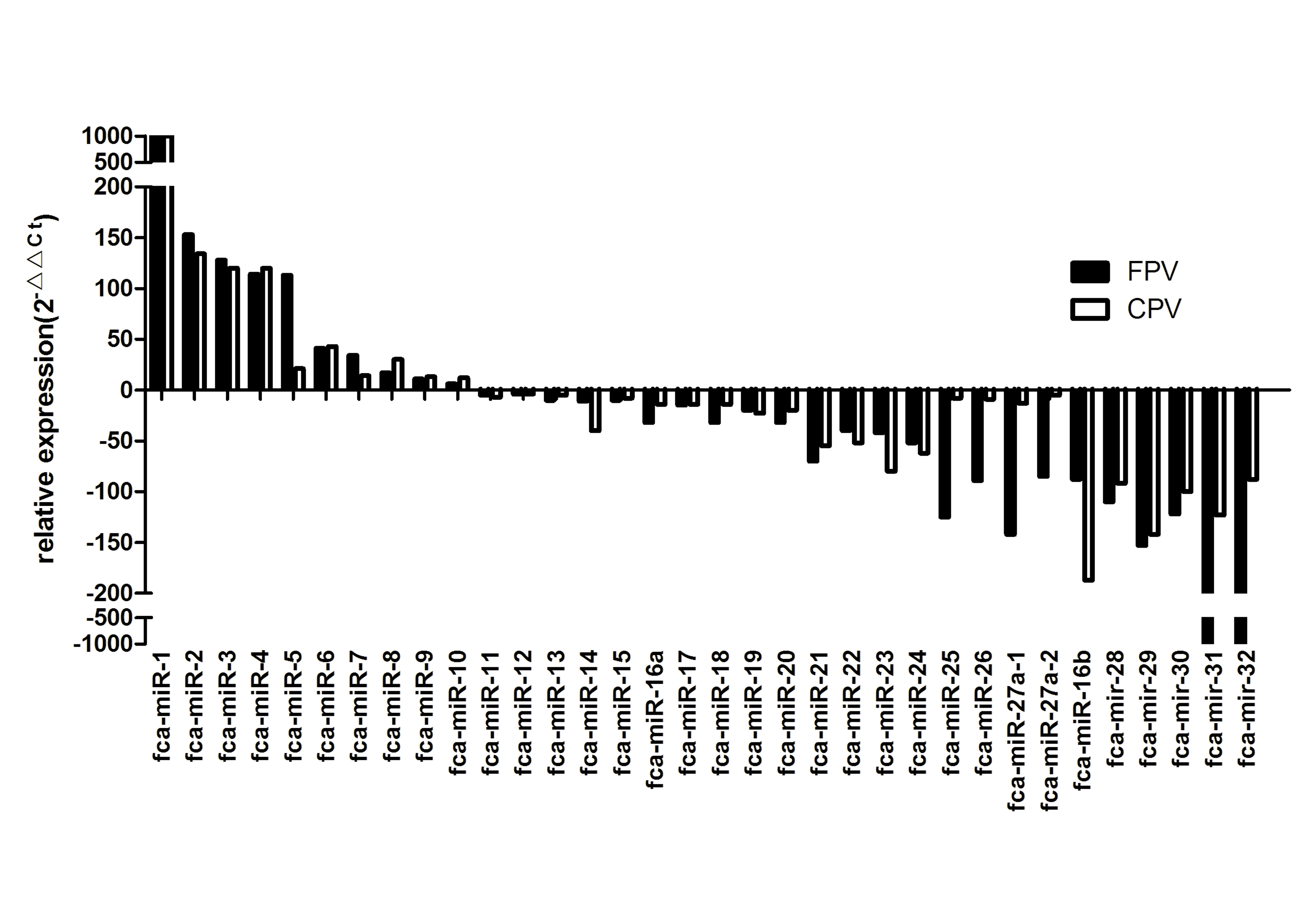

Supplement: S1 Fig — The relative expression was calculated using the 2-△△Ct method, and values greater than 1000 or less than -1000 are presented as 1000 or -1000. (TIF) [file pone.0185698.s002.tif]
